# Building a New Framework for Urban Parking Facilities Research with Quality Improvement: The Case of Chongqing, China

**DOI:** 10.3390/ijerph20010607

**Published:** 2022-12-29

**Authors:** Shifang Liu, Shaohua Tan

**Affiliations:** 1 Faculty of Architecture & Urban Planning, Chongqing University, Chongqing 400030, China; 2 Key Laboratory of Ministry of Education of Construction and New Technology in Mountainous Town, Chongqing 400030, China

**Keywords:** China, parking facilities, environmental quality improvement, quality index, improvement strategy

## Abstract

People-oriented development has become the main theme of China’s current social development, and the construction of various urban infrastructure has shifted from a focus on functionalism to a continuous pursuit of service quality. As an essential infrastructure for urban transport, urban parking facilities have an impact on pedestrian experience and landscape appearance based on the provision of parking functions. Therefore, this study is oriented to improving the quality of parking facilities, proposes a research framework of parking facilities based on meeting functional demand and service quality, and constructs a quality index to evaluate the quality of parking facilities, which includes three dimensions of evaluation indexes: pedestrian space impact, environmental space impact, and demand matching. By analyzing the current characteristics of urban parking facilities and measuring their quality index (6.5), the study finds that while satisfying the basic function of parking demand, it brings a negative impact on the pedestrian experience and the overall urban landscape appearance of the city. Motivated by this, this study proposes strategies to improve the quality of parking facilities: demand matching, spatial synergy, and environmental design to address parking difficulties, while injecting different ideas for future value orientation of parking facility planning and construction.

## 1. Introduction

Since 1978, China has experienced 40 years of rapid reform and opening up, with rapidly increasing levels of urbanization and people’s material living standards, and motor vehicle travel has become the most influential mode of transport for people’s daily travel. According to statistics, the number of car ownership in mainland China reached 280 million by the end of 2020 (and has exceeded 300 million by the end of 2021), which has exceeded the United States (270 million), Japan (78 million), Germany (52 million), and other developed countries, ranking first in the world. The rapid increase in the number of cars has brought about radical changes in the productive lives of people. However, while people enjoy the convenience brought by car travel, they also suffer from a series of derived problems, the most influential of which is the problem of difficult parking. In order to solve such problems, countries around the world, governments at all levels continue to invest greater efforts to build parking facilities to better meet the demand for parking. The continuous construction of parking facilities, while serving parking, has led to the deterioration of the living environment and the fragmentation of the environmental landscape, and air pollution and other problems have intensified [1].

In response to the numerous problems of urban parking, scholars around the world have conducted extensive research. As urban transportation infrastructure has become particularly mature due to the early modernization process in developed countries, their research has mainly focused on improving traffic efficiency and optimizing park-and-ride systems with the goal of further optimizing the travel structure and efficiency. The development of smart parking systems has become an industry hotspot in recent years. Through machine learning, program development, and cell phone program application, the smart parking system is continually improved and supplemented to facilitate drivers’ parking [2]. Farhana, B., focused on the impact of spatial location of parking facilities on park-and-ride, and used a model of facility location optimization to improve overall transfer efficiency, thus leading to changes in travel patterns and structures, and gradually strengthening the proportion of public transportation [3]. Some scholars focus on the goal of sustainable transportation development, based on the zoning method of Tyson polygon for the spatial distribution of public parking spaces to establish the optimal parking location to reduce environmental pollution (control CO_2_ emissions) [4]. Meanwhile, urban parking is a major link in the urban transportation system and has an essential influence on the different traffic behaviors in the system. For example, the ease of parking directly affects the smoothness of traffic operation and the overall speed, distance, and experience of other traffic in the network. In turn, this effect is also present. Jin Cao et al. modeled the transition matrix of parking states to assess the impact of parking usability on dynamic traffic and the impact of dynamic traffic conditions on parking convenience [5]. At the same time, since the construction and development of parking facilities is deeply related to urban processes, and most developing countries are still in the phase of rapid urban development, most studies on urban parking have focused on typical cities in developing countries. For example, Ismiyati, for the city of Semarang, Indonesia, proposed a zoning-based integrated parking solution, which made a plan for the overall arrangement of the facility space in terms of parking rates, land value, location, and accessibility [6]. The use of parking facilities is evaluated by determining the parking performance index through four indicators: parking demand–capacity ratio, search parking time, walking time, and parking cost [7,8]. The shared parking scheme, a current solution to the parking problem proposed by researchers, is to share the demand for urban parking by making dedicated parking lots public during specific hours [9]. Similarly, some scholars have proposed reducing parking demand in certain areas by increasing motorized travel and increasing the complexity of urban streets to obtain a “minimum demand” for urban parking [10]. As can be seen, such studies also aim to solve parking problems in local cities and are of great practical importance.

In contrast, related research by Chinese scholars has focused on solving parking problems, promoting traffic efficiency and parking convenience, and has started to develop in recent years in terms of management and maintenance, as well as market intervention. It mainly focuses on parking facility demand forecast [11], parking facility spatial layout [12], parking facility scale calculation [13], parking facility management and maintenance [14], and parking facility operation mechanism [15], which plays an essential role in research and practice to solve the parking problem. Meanwhile, Chinese scholars have also demonstrated that drivers create additional needs, such as parking security, when basic parking needs are met through a study of bicycle parking environments on university campuses [16]. However, various problems in urban parking have been difficult to plan, construct, and manage due to large changes in urban parking demand, management difficulties, and prominent conflicts between people and vehicles. At the same time, some scholars have achieved great results in exploring parking problems from the object properties of parking facilities [17], but are still limited to the inherent functional properties of parking facilities without paying attention to the environmental elements and human–vehicle relationship related to parking facilities, resulting in the construction of parking facilities that bring damage to the urban wholeness. For example, in a pedestrian environment where people are frequently impacted by assisted living facilities, their proper layout, reminder signs, and scale control will reduce this impact [18]. Different forms of parking can also have different effects. Researchers have suggested that on-street parking, while more convenient, is still the most dominant form of parking that causes urban congestion and frequent safety problems [19].

Thus, this paper is the first to analyze the implications of the attributes of urban parking facilities from an economic perspective and establish the value orientation of improving the quality of parking facilities. The authors then construct a research framework based on satisfying functional needs and service quality. Finally, based on this study, the authors propose a planning strategy for quality improvement of parking facilities, focusing on the Yuzhong District of Chongqing, China.

The objectives of this research paper are:

(1) To propose the planning value of parking facilities from the traditional focus on functionalism to the concept of human and environmental quality enhancement;
(2) Constructing an evaluation system by functional demand and service quality, and proposing the quality index as a standard to measure the current service level of urban parking facilities;
(3) Propose strategies for the quality improvement of parking facilities: three aspects of urban parking demand prediction method, spatial layout of urban parking facilities, and environmental improvement of urban parking facilities.

The results of this study provide original ideas for solving current parking facility problems and improving parking facility planning. At the same time, it can also provide a different value basis for the construction of related urban public service facilities in the process of urban development and construction.

## 2. Establishing a New Research Framework

### 2.1. Connotation of the Properties of Parking Facilities

The field of economics classifies goods into public and private goods based on competition and exclusivity. Universally, goods that can be provided for the whole society and are neither exclusive nor competitive are called public goods [20]. In contrast, private goods are obviously competitive and exclusive [21]. The comparison shows that the difference between public and private goods is expressed in whether anyone’s use has an effect on other consumers. The competitive and exclusive nature of goods, however, is subject to transformation with time and changes in the external environment. Such items are then called quasi-public or quasi-private goods [22]. In general, there are three characteristics of such goods: firstly, such goods can be supplied by the government or by the market, and can be supplied by the government or by the market after government subsidies; secondly, such goods, when used by a particular consumer, necessarily result in a diminution of the rights of different consumers; thirdly, such goods have price exclusivity, that is, although these goods is offered to the whole community, only those who pay to consume it are entitled to it. Therefore, the attributes of goods can be classified as public goods, private goods, and quasi-public goods (quasi-private goods) by competition and exclusivity.

There are various types of parking facilities, and the subjects of their development and construction include government, enterprises, etc., resulting in parking facilities having both public goods attributes and commodity attributes (i.e., private goods attributes). An accurate grasp of the nature of urban parking facilities is relevant to how to play the role of parking facilities and maximize the benefits. Using public parking facilities as an example, firstly, they are provided for the entire society to guarantee parking rights for members of society; secondly, as the number of cars increases, the parking facilities become competitive, going from non-competitive at the beginning to competitive later due to parking tightness; finally, parking facilities have explicit consumption exclusivity, and users without spending power will not be able to enjoy the convenience of parking facilities. Thus, urban parking facilities can be judged as quasi-public/quasi-private goods, hereafter referred to collectively as quasi-public goods.

The two main problems reflected by the current parking facilities are focused on the mismatch between supply and demand and the low quality of facility services [23]. First, the mismatch between supply and demand is due to the fact that the current parking facilities cannot completely meet the demand for parking in the city, that is, the functional properties as quasi-public goods cannot be better realized. At the same time, the construction of numerous parking facilities has led to the fragmentation of urban space, which has had a negative effect on the urban landscape and landscape environment; moreover, bringing interference to the daily travel of urban residents, which affects their travel experience, i.e., the services attributed as quasi-public goods are not reflected [24]. Thus, the key to the planning and construction of urban parking facilities is to achieve their two main objectives of meeting the quality of goods and services functions.

**Figure 1:**
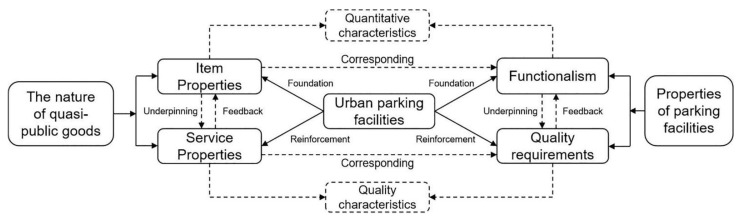
Connotations of attributes of urban parking facilities.

The basic function of an urban parking facility is to meet the urban parking demand and solve the urban parking difficulties. On the basis of safeguarding their essential functions, the quality improvement of a facility is inherent to a full understanding of the two main attributes of an urban parking facility, namely the object and service attributes. Moreover, these two attributes are a clarification of the quality improvement requirement, which is to improve the environmental benefits of urban parking facilities on the basis of safeguarding the basic needs of urban parking. The environmental benefits mentioned here mainly refer to the impact of parking facilities on the urban landscape and pedestrian travel. Therefore, quality improvement of urban parking facilities should be carried out in two ways: first, environmental improvement of individual parking facilities through their landscaping treatment, reasonable setting of entrances and exits, parking support construction, etc. Second, the environmental improvement of regional parking facilities, through the overall development of parking facilities in the area, reasonable parking space layout, and reducing the impact on the urban landscape to improve the quality of urban parking facilities [25] (Figure 1).

### 2.2. Study of Quality Improvement in Parking Facilities

The Planning Guidelines for Urban Parking Facilities (hereinafter referred to as the Guidelines) is a document issued by the Ministry of Housing and Urban–Rural Development of China in September 2015 which aims to scientifically promote the planning and construction of urban parking facilities, reasonably allocate parking resources, build an orderly parking environment, and guide traffic demand. The four principles proposed for the planning of parking facilities include the principle of demand management, the principle of coordination, the principle of resource conservation, and the principle of integrated management. These principles, in terms of their requirements and content, are centered on meeting demand and maximizing the use of resources to realize the benefits of urban parking facility construction. However, with the current high-quality requirements of urban development and construction, urban parking facilities should be considered more profoundly on the basis of meeting the basic functional requirements and the impact on the urban landscape environment and pedestrian travel experience [24] (Table 1).

**Figure 2:**
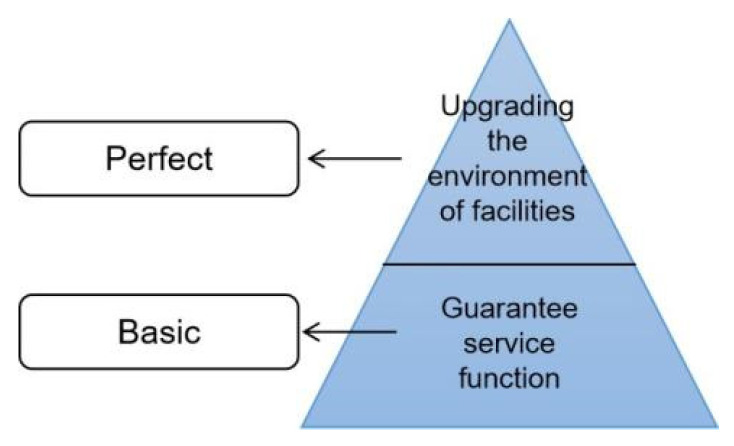
Logic of the parking facility quality improvement study.

Therefore, urban parking facilities should be planned on the basis of adhering to existing principles, focusing on the impact of the construction of the facilities on the urban landscape environment, and minimizing the interference with the daily travel of pedestrians. Motivated by this, this paper establishes the research logic of meeting the demand for urban parking facilities and overall quality improvement with the current environmental benefit enhancement perspective. Basic function: To meet the demand for parking in the city; quality improvement: to match the needs of the environment and people (Figure 2).

### 2.3. Quality of Construction Index: System for Evaluation of Parking Facilities Index

Based on the research framework developed in this study, to meet the planning requirements for quality improvement, this study proposes the concept of quality indexing, which is the establishment of an evaluation indexing system to improve the quality of parking facilities. The first is to evaluate the layout of parking facilities in terms of service level, spatial impact, and construction investment [26]; the second is to set evaluation indexes according to different groups such as parking users, parking operators, neighboring residents, and the government [27]. Combining with the existing studies, the authors selected three aspects of the evaluation indexes based on the connotation of the attributes of parking facilities, the impact of parking facilities on pedestrian space, the impact on environmental space, and the degree of matching parking demand, based on pedestrian and environmental demand [28,29] (Table 2).

## 3. Case Study in Chongqing, China

### 3.1. The Study Area

**Figure 3:**
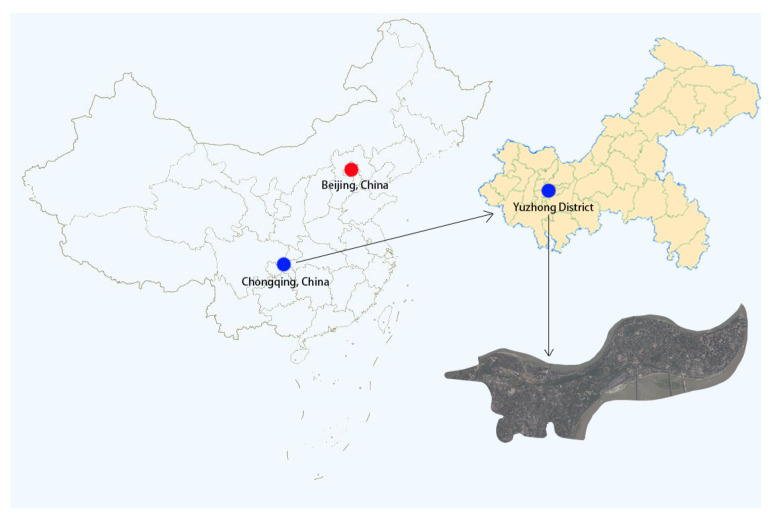
Location analysis of Yuzhong District, Chongqing, China.

The mountain city of Chongqing in southwest China was chosen as the case study for this study. According to Baidu Map’s 2021 China City Traffic Report, Chongqing ranked second in the list of the most congested cities in mainland China in 2021, and the density of parking construction in major cities ranked in the top ten nationwide. Meanwhile, Yuzhong District, as the core area of the main city of Chongqing, has multiple functions such as finance, commerce, and cultural tourism, and belongs to the traditional landscape protection zone, and it is representative to implement studies related to the enhancement of parking facilities [30] (Figure 3).

Therefore, there are three reasons for choosing Yuzhong District in Chongqing as the scope of the study: (1) Yuzhong District belongs to the old city of Chongqing, where the construction of parking facilities is relatively well established, and the study of it can more directly reflect the current problems of parking facilities; (2) Yuzhong District, as the core area of the main city, is the most densely populated road and business district in Chongqing [31]. The construction of parking facilities, while meeting the use function, has a greater impact on the overall environment and pedestrian experience; (3) Yuzhong District carries abundant urban functions, which can directly reflect the layout characteristics of parking facilities between different functional areas [32].

**Figure 4:**
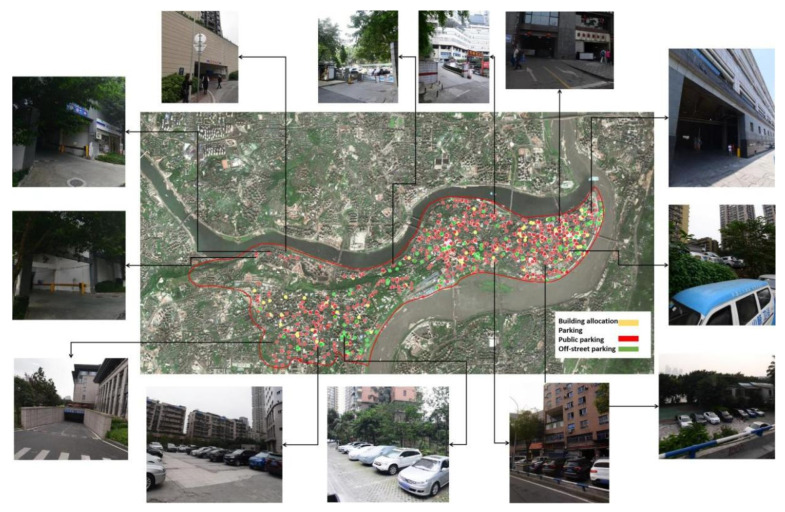
Selected locations of parking facilities in Yuzhong District.

According to the division of parking facility types in the 2015 version of the guideline, urban parking facilities can be broadly classified into three categories: building-assigned parking plenty, urban public parking plenty, and on-street parking spaces. According to this standard, the authors sorted out the parking facilities in Yuzhong District in 2020 and, combined with satellite maps and POI data (traffic facility data), a total of 917 parking facilities of three types were identified, including 680 public parking lots, 85 parking lots allocated to buildings, and 152 on-street parking lots (Figure 4 shows the distribution map of parking facilities in Yuzhong District).

### 3.2. Research Methods

In this study, field studies and questionnaire surveys were mainly used, and the quality assessment index model constructed in the previous section was combined to perform a comprehensive analysis of the study cases.

The spatial environmental characteristics of the parking facilities were obtained through field studies. The questionnaire was used to obtain basic information about respondents and the impact content of parking facilities. The questionnaire was split into a pedestrian version and a driver version for the different status of the respondents. The questionnaire process uses semi-structured interviews, firstly, through open-ended questions, to understand whether respondents can accurately participate in this survey. Second, the questionnaire was completed through a fixed set of questions. The questionnaire was divided into three parts, which were applied to both versions of the questionnaire. First, the basic information of the respondents was collected, including place of residence, gender, age, etc. Second, there is the impact of the content of the parking facility on the behavior of the respondents. Third, there is the respondents’ views on parking facilities.

#### 3.2.1. Pre-Survey

To better conduct this study, the authors performed a pre-experiment prior to the experiment. The pre-experiment selected six parking facilities in Jiangbei District of Chongqing, covering three types of parking facilities. The questionnaire and fieldwork were optimized through field interviews and photographs. The reasons for choosing Jiangbei District are (1) it belongs to the core area of the main city of Chongqing, like Yuzhong District; (2) it has the characteristics of a mountainous city; (3) it is densely populated with a dense flow of people and vehicles. Almost immediately, because of the impact of the current coronavirus epidemic, the authors chose the closer Jiangbei district for the pre-study.

The pre-study was conducted on 4 May 2021 (Tuesday), using the same methods and steps as the formal research, interviewing 30 people on site, distributing 26 questionnaires, and collecting 26 copies. Through the pre-study, the authors found two problems with the content of the questionnaire: (1) the personal characteristics of the respondents were ignored and some of them did not meet the interview requirements; (2) the content of the questionnaire only focused on the parking environment and did not cover the impact of parking facilities on the respondents. Therefore, the authors also adjusted the content of the questionnaire.

#### 3.2.2. Field Research

**Figure 5:**
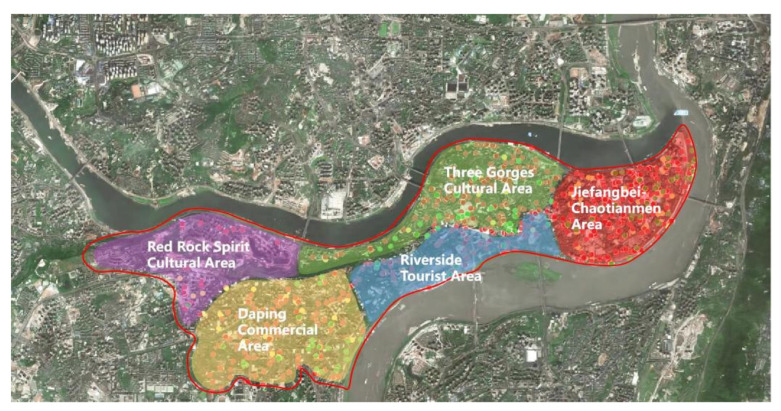
Diagram of the functional zoning of Yuzhong District, Chongqing.

Based on the identified 917 parking facilities, the authors finally selected 32 parking facilities for field research, taking into account the division of the 5 functional zones in Yuzhong District (Figure 5). Among them, there are 8 in the Jiefangbei–Hongyadong area, 6 in the Three Gorges Culture area, 5 in the Riverside area, 6 in the Red Rock Spirit Culture area, and 7 in the Daping Commercial area. The authors conducted a four-day field study in June 2021, on weekdays (9–10 June 2021) and weekends (12–13 June 2021), with two days on weekdays and two days on weekends (the weekend of which was the Chinese traditional festival of Dragon Boat Festival).

A total of 128 photographs were taken and 208 valid questionnaires were collected during the field study. The authors interviewed a total of 242 people. In total, 211 questionnaires were distributed, and 208 valid questionnaires were collected (Three of the questionnaires were filled in by tourists.) Of the 242 respondents, 31 people (foreign tourists) clearly indicated the identity of the respondents, thus no questionnaire was administered.

In the live photos, the entrance and exit points of the parking facilities, the site conditions, the surrounding environment, the relationship with dynamic traffic, and the relationship with pedestrians were recorded according to the five functional areas divided within the study area. From the 32 parking facilities, the authors selected 9 typical parking facilities to summarize the information. (Table 3 shows the information of 9 typical parking facilities).

The valid questionnaires included the pedestrian version (100) and the driver version (108). The pedestrian version mainly sets questions about the impact of the parking facilities that pedestrians pass through on their daily journeys, while the driver version mainly sets questions about the satisfaction of parking needs and the feeling of the parking process.

### 3.3. Data Analysis

#### 3.3.1. Analysis of the Questionnaire Information

First, SPSS descriptive statistics were used to determine the basic information of the respondents.

Second, the SPSS tool was used to perform a correlation analysis on the influence content of parking facilities to determine the relation of each influence element. Pearson correlation coefficient was used for the correlation analysis, which can reflect the degree of influence of each pair of independent variables on the dependent variable, and is measured from −1 to +1, reflecting the direction and degree of shift in the trend between two variables, with 0 indicating that the two variables are not correlated, positive values indicating positive correlation, and negative values indicating negative correlation, with larger values indicating stronger correlation. The formula for the calculation is as follows [33]:

(1) $$R=\frac{\sum_{i}^{n}\left(X_{Z}\bar{X}\right)\left(Y_{Z}\bar{Y}\right)}{\sqrt{\sum_{i}^{n}{\left(X_{Z}\bar{X}\right)}^{2}}\sqrt{\sum_{i}^{n}{\left(Y_{Z}\bar{Y}\right)}^{2}}}$$

Note: *R* is the Pearson correlation coefficient; *n* is the number of samples; *Xz* and *Yz* are the sample numbers corresponding to the variables *X* and *Y*; $\bar{X}$ and $\bar{Y}$ are the mean values of the samples *X* and *Y*, respectively.

#### 3.3.2. Quality Index Analysis of Parking Facilities

(1) Impact of parking facilities on pedestrian space

The impact of parking facilities on pedestrian space is reflected in the effects caused by the daily movement of residents in its vicinity [18]. Previous studies have found that the number of parking facilities is 1–3 in general, and the number of entrances and exits to parking facilities is 1–3 within the daily activities of pedestrians, 77% and 86%, respectively. The main factors affecting pedestrian travel are the location and number of entrances and exits to parking facilities, as well as the number and size of parking facilities. Therefore, the evaluation scope is centered on the residential community, an area range of 500 m radius was selected (500 m range is based on the distance suitable for walking in Chinese landscape design standards, the same below), and the evaluation function of pedestrian travel impact is constructed as follows.

E = (B/APQ)·K(2)

Note: E is the value of the evaluated travel experience. The larger the value of E, the less impact parking facilities have on the travel experience of pedestrians.

A is the number of parking entrances and exits.

B is the distance between the entrance and exit of the parking lot.

P is the number of parking facilities in the area.

Q is the size of the demand for parking facilities, which is the number of available parking spaces.

K is the overall evaluation correction index for parking facilities. The effect of parking facilities on pedestrian space is not only influenced by the characteristics of the parking facilities themselves but is also directly related to the location of the facilities. In other words, if the object of study is located in the center of the city, the effect is relatively large; if the object of study is located in an area far from the city center, the effect is relatively small. As a result, parking facilities with the same characteristics in different locations will have different impacts. The city center is determined by the city group-level commercial center, and the straight-line distance from the parking facility to the city center is measured by Baidu map, which is divided into four intervals: within 500 m, 500~1000 m, 1000~1500 m, and above 1500 m, and the corresponding correction coefficients are determined as 1.00, 0.85~0.95, 0.75~0.85, and 0.55~0.65.

(2) Impact of parking facilities on environmental space

The impact of parking facilities on the urban landscape is due to the number of parking facilities in the area, the location and number of entrances and exits, and the type of parking facilities. The number of parking facilities will vary depending on the size of the chosen area range. In this paper, the scope of the research evaluation is defined as an area range with a radius of 500 m with a certain urban landscape as the center (the urban landscape selection criteria are mainly based on scenic spots, natural greenery, and wind and landscape coordination). Therefore, the evaluation function for the impact of the urban landscape is constructed as follows:

I = (B/PA)·N·K(3)

Note: Letter I is an impact assessment of the value of the landscape. The larger the value of I, the lower the impact of parking facilities on the urban landscape.

B is the distance between the entrance and exit of the parking facility.

P is the number of parking facilities in the area.

A is the number of parking entrances and exits.

N is the type of parking facility. When N is surface parking, the value of N is 0.2; when N is on-street parking, the value of N is 0.4; when N is underground parking, the value of N is 0.8. The authors argue that underground parking has the least impact on the cityscape, while surface parking has the most. K is the modified index for the overall evaluation of the parking facility environment (based on the same K value as above).

Different forms of parking facilities have different impacts on the urban environment, so they are assigned separately. While surface parking lots are excellent for parking behavior and ease of vehicle access, they take up a large amount of ground space and their impact on the urban environment is most drastic and harmful; underground parking lots, where parking spaces are located primarily underground, leave only entrances and exits above ground, so their environmental impact is minimal; overhangs, surface parking structures, cover relatively small areas, and their impact on the environment is primarily reflected in their harmony with their surroundings and coordination of the surrounding environment. Eventually, the environmental impacts of the three types of parking facilities are assigned [34] (Table 4).

(3) Matching of parking facilities with parking demand

Parking demand forecasting, which converts the number of all types of motor vehicles in a city into equivalent tiny car ownership and the number of parking spaces in all parking plenty, can visually reflect the relationship between parking demand and supply [26].

S = Pt/Qt(4)

Note: S indicates the level of parking demand satisfaction; Pt indicates the number of regional parking facilities supplied in year t; Qt indicates the number of regional parking requirements in year t. The parking capacity at year t = the projected parking demand at year t, S = 1. In other words, the larger the difference between the values of S and 1, the less desirable the satisfaction of the parking demand in the area. Either undersupply or oversupply will cause S to deviate from 1.

(4) Comprehensive evaluation of parking facilities

Construct a composite function of parking facilities in terms of parking demand, environmental impact, and pedestrian impact to determine the comprehensive evaluation results.

F = {E,I,S}(5)

Among these, there is a positive correlation between E and I, that is, poor walking experience in actual measurements is followed by a large effect on the environmental landscape. Excellent walking experience has a relatively minor impact on the environmental landscape. The S-value is used as the satisfaction level of parking demand and the overall evaluation results are corrected. At the same time, as in the environmental impact assessment I, the effects on air pollution, noise pollution, plant damage, etc., are not covered for the time being, and the overall impact on the appearance of the landscape is mainly considered.

The larger the value of the final composite function result F, the higher the overall evaluation of the parking facility. In turn, the smaller the F value, the lower the rating of the parking facility (The F value is measured using a ten-point scale) (Table 5).

## 4. Results

### 4.1. Basic Information about the Interviewee

A total of 208 questionnaires were obtained for this study and 208 respondents of interest to the study were identified, including 100 pedestrians and 108 drivers. For pedestrians, respondents were identified based on the fact that they lived within 500 m of a parking facility and had a daily walking habit (including commuting, walking, etc.). For drivers, respondents were identified based on the occurrence of defined parking behaviors and frequency of parking at least three times per week, which established them as eligible respondents.

#### 4.1.1. Basic Features

Analysis of descriptive statistics from SPSS: 58% of pedestrians were male and 42% female. Moreover, the age structure was concentrated between 23 to 35 years old (48%) and 36 to 50 years old (25%). Of the drivers, 71.3% were male and 28.7% were female. Moreover, the age structure was concentrated between 23 to 35 years old (58.3%) and 36 to 50 years old (36%). Both pedestrians and drivers were found to be predominantly male, with age structures consistent with behavioral profiles. See Table 6 for basic information about the respondents.

Meanwhile, the results show that (1) in general, the occupational information of the respondents shows that Yuzhong District is the core area of government functions and commercial trade, and more than 70% of the respondents are government employees and personnel of enterprises and institutions; (2) drivers: most drivers have fixed parking places and can satisfy their own parking needs; (3) pedestrians: pedestrians walk for about one hour a day, which is closely related to the construction of the Yuzhong District Mountain City Trail.

#### 4.1.2. Behavioral Characteristics of the Driver

**Figure 6:**
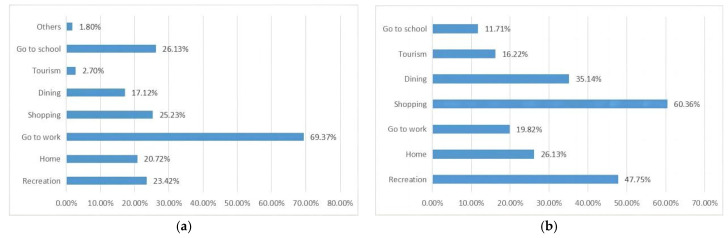
Parking purpose on weekdays and holidays. (**a**) Purpose of parking on weekdays, (**b**) Parking purpose on weekends and holidays.

**Figure 7:**
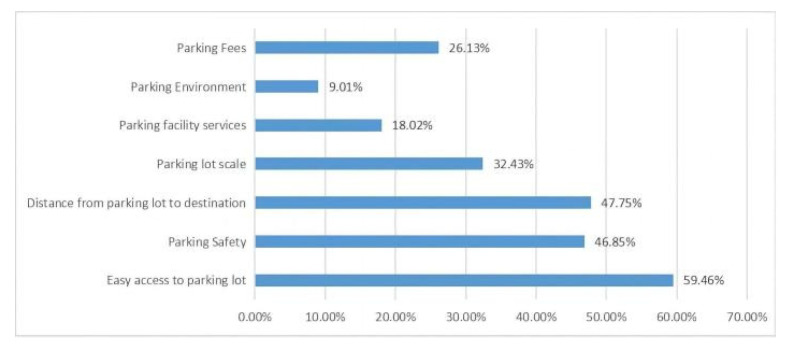
Considerations for choosing a parking facility.

**Figure 8:**
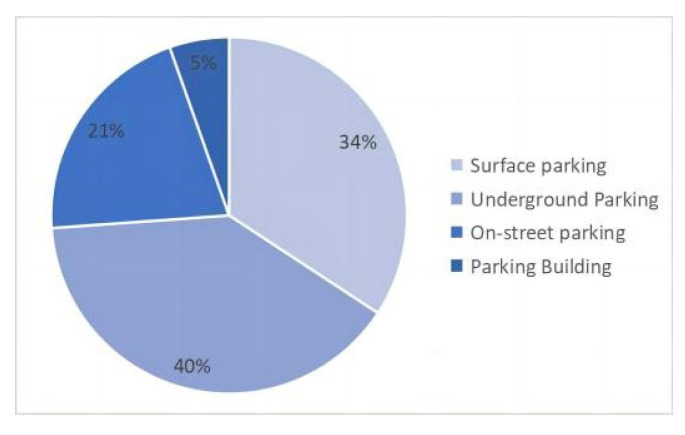
Proportion of parking facility options.

**Figure 9:**
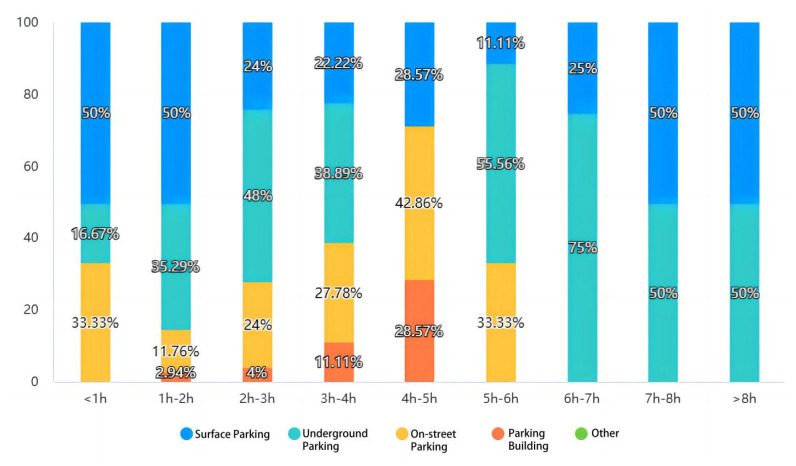
Parking hours correspond to different parking facilities.

It has been shown that drivers’ choice of parking facilities is based on the walking distance from their destination, the strength of accessibility, and parking safety [15]. Based on this, the study was conducted with SPSS descriptive statistical analysis in terms of parking purpose, duration, choice of parking facilities, and negative impact of parking facilities. It was found that:

(1) Purpose of parking (Figure 6). Statistically, the purpose of parking on weekdays is mainly to go to work (69.37%), and the purpose of parking on weekends/holidays is mainly shopping (60.36%), entertainment (47.75%) and gathering (35.14%). Parking purposes highlight the prominent function of Yuzhong District as a commercial tourism and cultural center in Chongqing.

(2) Parking facility selection (Figure 7 and Figure 8). Statistics show that more than 70 percent of the population opt for surface and underground parking facilities. The impact of surface and underground parking facilities on pedestrian access and landscape integrity is greatest.

(3) The length of parking in different parking facilities (Figure 9). Statistically, about 70% of drivers park between 1–4 h. Due to the lengthy parking duration, surface parking lots and underground parking lots have become the main choice (more than 70%). Both types of parking lots are safer and easier to manage than other parking lots. At the same time, in the parking behavior within 1 h, most people choose on-street parking. The longer it takes, the more drivers choose surface and underground parking.

#### 4.1.3. Description of Pedestrian Walking Environment

This study aims to analyze the impact of parking facilities on the walking experience of pedestrians through the perspective of pedestrians, including the impact on pedestrian walking and the impact on the environment. Therefore, the questionnaire was designed to determine the environmental impact of walking by the number of parking facilities and the number of entrances and exits to the parking facilities.

**Figure 10:**
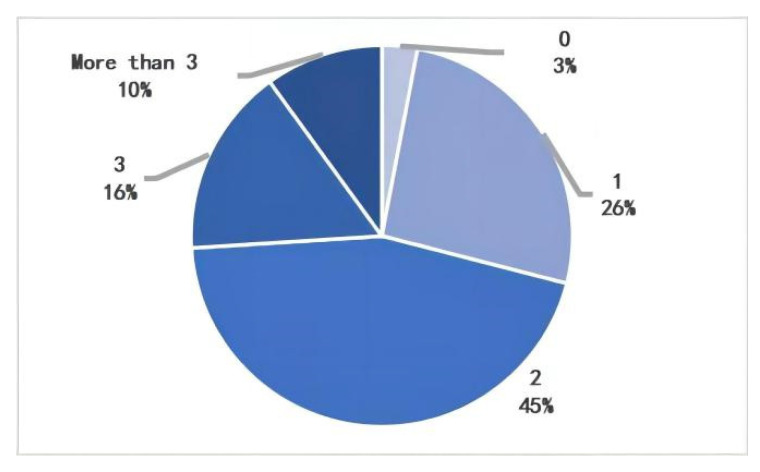
Number of parking lots passed on the walk.

**Figure 11:**
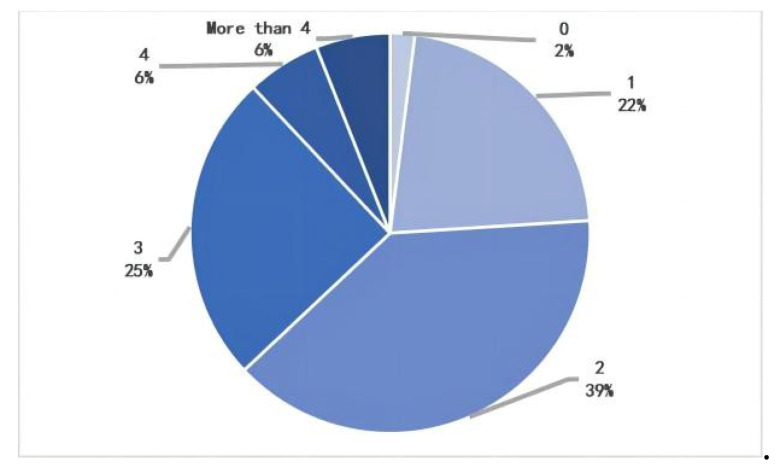
Number of parking entrances and exits on the walk.

Statistically, 26% of pedestrians pass through 1 parking lot, 45% pass through 2 parking lots, and 26% pass through 3 or more parking lots during their daily walk. Correspondingly, the statistics show that 22% of pedestrians pass through 1 entrance/exit, 39% pass through 2 entrances/exit, 25% pass through 3 entrances/exit, and 12% pass through 4 or more entrances/exit. The study found that more than 50% of pedestrians would pass through 2 or more parking lots, corresponding to additional entrances and exits to parking facilities (Figure 10 and Figure 11).

### 4.2. Correlation Analysis of Impact Elements of Parking Facilities

This study focused on the impact of parking facilities on the spatial environment, and therefore conducted a correlation analysis of the impact elements of parking facilities on the basis of driver choice of parking facilities and the impact of parking facilities on the pedestrian environment.

#### 4.2.1. Factor Analysis of Drivers’ Choice of Parking Facilities

Based on the questions listed in the questionnaire, SPSS correlation analysis was performed on three variables: the environmental factors respondents were concerned about for parking facilities, the basis for respondents’ choice of parking facilities, and the top factors for respondents’ choice of parking facilities.

From the table (Table 7), we can see that the correlation analysis was used to study the correlation between the environmental factors that respondents care about parking facilities, the basis for choosing such parking facilities, and the top factors for choosing parking facilities. The Pearson correlation coefficient shows a correlation of −0.243 between the environmental factor of the parking facility and the primary factor in the choice of the parking facility, indicating a significant negative correlation; the correlation coefficient between the basis for choosing such a parking facility and the primary factor for choosing a parking facility was 0.207, indicating a significant positive correlation. The analysis results show that drivers are more concerned with the basic functions of parking facilities, and on this basis, parking convenience and safety became the most significant factors.

#### 4.2.2. Analysis of Factors Affecting Pedestrians by Parking Facilities

Based on the questions listed in the questionnaire, SPSS was used to analyze the correlation between the number of parking lots the respondents passed through on their walks, the number of parking entrances and exits, whether the parking facilities were clearly marked, and whether the respondents thought the parking facilities would have an impact on their walks.

From the table (Table 8), correlation analysis was used to investigate the correlation between the number of parking lots the respondents passed on their walks, the number of entrances and exits of the parking lots the respondents passed on their walks, whether the parking lots were obviously marked, and whether the respondents thought the parking facilities had an impact on their walks. Pearson correlation coefficient was used to indicate the strength of the correlation, which the analysis shows.

(1) The correlation coefficient between the number of parking lots that respondents pass on their walks and the perceived impact of parking facilities on walking is 0.513, and is significant at the 0.01 level, thus indicating a significant positive correlation.
(2) The correlation coefficient between the presence of distinctly marked parking lots and the perceived impact of parking facilities on walking was 0.437 and showed a significant correlation at the 0.05 level, thus indicating a significant positive relationship.
(3) The correlation coefficient between the number of entrances and exits of the parking lots that the respondents pass through on their walks and the perceived impact of parking facilities on walking is −0.324, which is significant at 0.05 level, thus indicating a significant negative correlation.

The result is that the greater the number of parking facilities, the greater the impact on pedestrian walking. In turn, significant parking facility signage has a correspondingly weaker effect on pedestrian walking.

### 4.3. Characteristics of Parking Facilities

**Figure 12:**
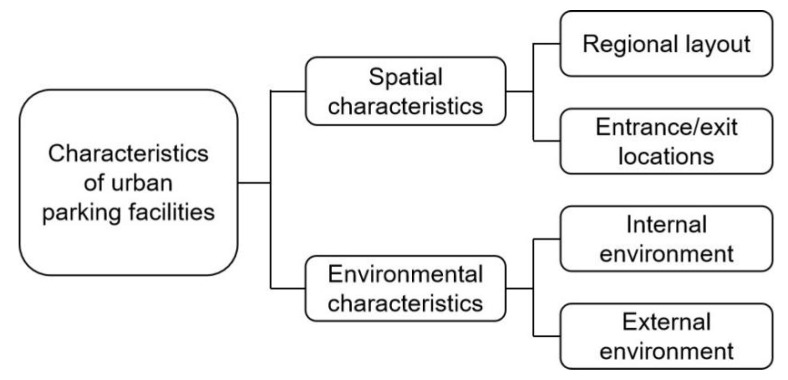
Connotation of urban parking facilities features in Yuzhong District.

Spatial environmental characteristics of parking facilities in Yuzhong district were observed and evaluated through field studies. The authors counted the features of the location of the parking facility, the surrounding environment of the facility, and information about the entrance and exit. Parking facilities in Yuzhong district fully reflect the peculiarities of mountainous cities. First, mountainous cities have tight land resources and make full use of marginal areas of urban development and construction, as well as poorly available space. Second, they have a distinct regional character, with large parking needs and traffic. Third, the layout of the facilities has led to an impact on the landscape appearance of the mountain town and has interfered with pedestrian travel within the city. The features of parking facilities in Yuzhong district are classified and organized into two categories: spatial features and environmental features. The spatial characteristics mainly consider the impact of parking facilities on the spatial environment of the urban area, while the environmental characteristics mainly focus on the environmental quality condition of the parking facilities themselves [35,36] (Figure 12).

#### 4.3.1. Spatial Features of Parking Facilities

**Figure 13:**
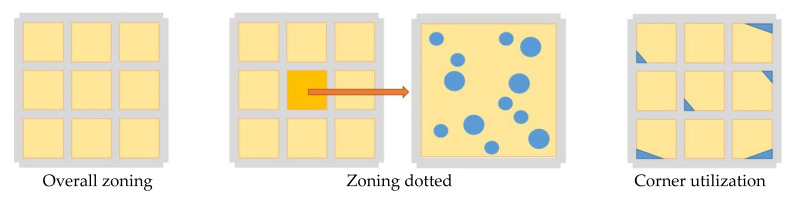
Characteristics of the regional layout of urban parking facilities.

**Figure 14:**
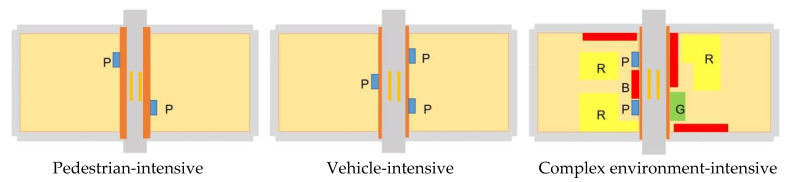
Location characteristics of entrances and exits to urban parking facilities. Note: P is for parking facilities; R is for residential use; B is for commercial use; G is for green space.

The spatial characteristics of urban parking facilities in Yuzhong District are expressed in the regional layout and the location of entrances and exits (i.e., external connections) (Figure 13 and Figure 14). The characteristics of regional layout are: ① overall zoning: the distribution of parking facilities has significant zoning characteristics, and the types and distribution of parking facilities in different zones are different; ② zoning dotted: within the zone, due to land resources, accessory buildings, etc., there is a scattered, free dotted layout; ③ corner utilization: the facilities are concentrated in the corners of urban construction and development, and the land is completely utilized.

The entrance/exit location of parking facilities, which is the external connection channel of parking facility space, not only has a close relationship with whether drivers can park conveniently, but also has an essential impact on the dynamic traffic and pedestrian population in the city [37]. After analysis, the location of entrances and exits have the following characteristics: ① pedestrian-intensive: located in areas with strong pedestrian flow, which affects pedestrian travel; ② vehicle-intensive: located on roads with strong traffic flow, where the entry and exit of vehicles affects dynamic traffic; ③ complex environment: the location of entrances and exits is determined by the location of parking facilities, which leads to a variety of land types around such parking entrances and exits, covering residential, commercial, and green areas, etc., which affect the normal operation of urban functions.

#### 4.3.2. Environmental Features of Parking Facilities

**Figure 15:**
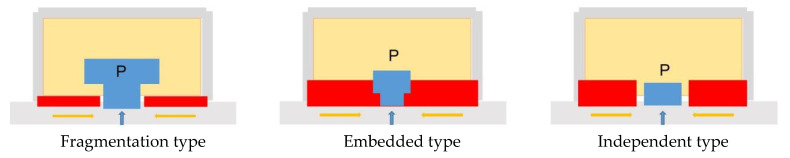
Characteristics of the external environment of urban parking facilities.

**Figure 16:**
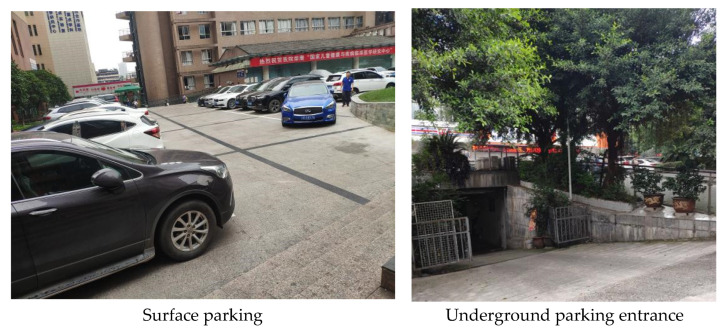
Characteristics of the internal environment of urban parking facilities.

The environmental characteristics of parking facilities mainly consider the impact on the urban landscape environment, analyzed from the external and internal environment of the facilities [36] (Figure 15 and Figure 16).

The external environment of an urban parking facility presents the following characteristics: ① Fragmentation type: the installation of parking facilities divides the originally unified and continuous interface, forming a barrier to passage or a gap in the environment. ② Embedded type: the installation of parking facilities is in the same interface with the street facade, forming a unified and continuous whole with additional functional entrances. ③ Independent type: the setting of parking facilities and the surrounding environment are independent of each other, usually appearing in surface parking lots, with separate sites and entrance/exit settings.

The internal environment of an urban parking facility, that is, the environmental quality of the parking facility itself, is shown in the form of landscaping and paving. Through research, it has been found that the greening of the interior environment of a parking facility is closely related to the form of the parking facility. The way the surface parking lot is constructed has an impact on the urban landscape, while its site environment plays an essential role in the formation of urban microclimate [31,38]. For underground parking garages, whose parking spaces are hidden below ground and are limited by light, ventilation, and technology, they are usually decorated by greening the entrances and exits, thus enhancing their landscaping effects.

### 4.4. Quality Index of Parking Facilities

This study establishes a research framework for overall environmental benefit enhancement and constructs a set of evaluation metrics for urban parking facilities in terms of quality improvement. By analyzing the overall parking facilities in Yuzhong District, Chongqing, China, and considering the evaluation results of three elements (drivers, pedestrian crowds and urban landscape environment), the evaluation method achieves a combination of qualitative and quantitative approaches, combining the unquantifiable impact elements of the environment with objective evaluation data of the parking facilities. The results show that the overall environmental evaluation score of parking facilities in Yuzhong District, Chongqing is 6.5, which can meet the basic function of parking demand better but has a greater impact on pedestrian travel and urban landscape environment. It can be seen that there are more problems in the planning and construction of parking facilities in Yuzhong District, Chongqing, in terms of dealing with the relationship with the spatial environment and pedestrian travel.

## 5. Discussion

### 5.1. Environmental Constraints on Parking Facilities in Yuzhong District

Located on the Yuzhong Peninsula in Chongqing, Yuzhong District has typical mountainous urban terrain and is the confluence of the Yangtze River and the Jialing River. Connections to additional urban areas are made primarily by cross-river bridges. At the same time, Yuzhong District serves as a business office, tourism and leisure, cultural heritage, and other essential functions, and is Chongqing’s calling card, which additionally leads to increased traffic pressure, including vehicular traffic and also pedestrian traffic. Finally, Yuzhong District, as an ancient urban area in Chongqing, has also contributed to the problems of high traffic pressure, difficult environmental coordination, and intensified conflicts between people and vehicles to some extent due to its compact land, elevated population density, and relatively dense road network construction. What needs to be objectively recognized is that Yuzhong’s own characteristics, while contributing to the poor environmental benefits of current parking facilities, have also created distinct urban landscape features in Yuzhong. In future urban renewal and urban design processes, the focus will be on rational use of spatial resources and integrating the advantages of land, people, transportation, and landscape. The overall environmental enhancement of Yuzhong District will go further.

### 5.2. Quality Improvement Strategies for Parking Facilities

Analyzing the current nuisances faced by urban parking facilities in Yuzhong District and guided by a different research framework, this study finds that attention should be focused on three areas in the planning of parking facilities.

#### 5.2.1. A Way to Match the Demand for Parking in Cities

**Figure 17:**
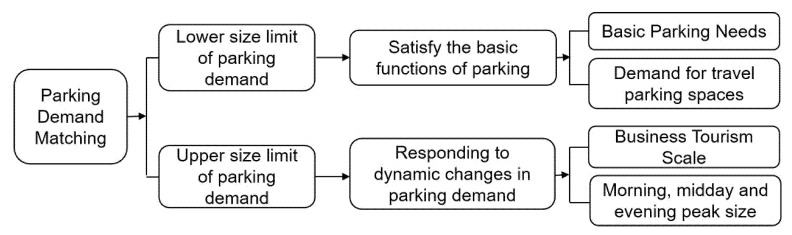
Parking facility demand forecasting methodology.

Parking facilities are planned on the basis of parking demand projections, but to avoid the vicious circle created by meeting parking demand. Therefore, the demand forecast for parking facilities should follow the principle of “top line and bottom line”. In determining the minimum supply, the upper limit of the supply should be tightly controlled. At present, parking demand forecasting is based on the parking generation rate model (overall urban parking demand, zoning demand) and static traffic generation rate model [39]. Based on this, the authors believe that the parking demand forecast must be able to cope with the dynamic changes in parking demand, i.e., by reasonably estimating the peak size of the tourist peak period and daily morning, midday and evening, the maximum parking size that can be carried is analyzed as the “upper limit” of the parking demand forecast. Secondly, the “lower limit” of the parking demand forecast should be combined with the basic demand of urban parking and the scale of travel vehicles (Figure 17).

#### 5.2.2. Spatial Synergy of Parking Facility Layout

The synergy of the spatial distribution of parking facilities should not only be able to reasonably match the parking demand of critical nodes within each significant area of the city but also avoid the impact on the urban landscape [40]. First, the parking areas should be divided according to the functional zoning of the city, with reasonable scatter among the zones and reasonable clustering within the zones. Based on the subsidiary characteristics of the parking facilities, the parking zones will be established according to the city’s commercial center, tourism center, office center and leisure center. At the same time, by coordinating the parking facilities inside and outside the area, the maximum benefit of the parking facilities can be realized on the basis of the unified environmental appearance [41]. Second, parking facilities are reasonably laid out around core functional units by incorporating the functional positioning of the area. Third, key units within each z26ing facilities should not affect the integrity and continuity of the landscape. Therefore, based on a reasonable prediction of the parking scale, spatial layout systems for parking facilities are developed to form spatial distribution patterns in slices, districts, and intra-districts. The spatial layout system of urban parking facilities in Yuzhong District has been gradually improved, in line with the requirements of serving the main body, taking into account individuals while not affecting the urban landscape.

#### 5.2.3. Environmental Design of Parking Facilities

The environmental design of parking facilities is key to realizing the value of quality improvement and is the most critical way to harmonize parking facilities with the urban environment. In the planning sense, it is a necessary means to achieve the overall protection and utilization of the mountainous townscape [42]. The environmental design of parking facilities incorporates both internal and external environments. Among them, the interior environment relies on technical landscaping combined with interior pedestrian access, disabled access, and security signs for the car park, and enhances the landscape effect by adding green vegetation, creative landscaping vignettes, and floor-to-ceiling pavement designs. Concerning external environmental enhancement, primarily for parking entrances and exits, and areas connected to the external environment, landscaped environments are shaped to achieve perfect integration with the surrounding environment. At the same time, the impact on passing pedestrians and dynamic traffic is reduced by setting up obvious entrance and exit signs, and stop signs that quickly guide motorists to a smooth stop.

## 6. Conclusions

This study reflects the fact that, under existing planning concepts, the construction of urban infrastructure remains focused on its own function, regardless of its relation to the surrounding environment. Therefore, by analyzing the connotation of “goods” and “service” attributes of urban parking facilities under the guarantee of quality demand, we emphasize that parking facilities should pay attention to the improvement of quality demand on the basis of satisfying functional demand. It also proposes a current framework for planning urban parking facilities, combining the basic guarantee of satisfying parking demand and environmental enhancement to meet the requirements of urban landscape, and proposes corresponding planning strategies in three aspects: the change of the method of urban parking demand prediction, the spatial layout of urban parking facilities, and the environmental enhancement of urban parking facilities. Of course, the quality of parking facilities is not only focusing on pedestrian and vehicular traffic in planning, and the relationship with the urban environment, but also involves the management and charging of parking facilities, the operation of parking facilities, social benefits, and numerous other aspects, which is a complex social matter. Therefore, the attempt of this paper is only to provide a different way of thinking for peer experts and scholars, focusing on the quality implications of planning in future planning and construction, as well as the creation of a wonderful human life.

Note: (1) Unless otherwise noted, the images and tables in the text were drawn by the author. (2) All equations in the methodology of this study were developed by the authors themselves.

## Figures and Tables

**Table 1:** Planning values for urban parking facilities.

Basic Values	Planning Objects	Service Users	Planning Concept	Planning Content
Functionalism	Parking facilities	Motor vehicles	Meeting urban parking needs	Parking development targets and parking facility planning are based on car ownership, combined with economic and social development and urban transport development objectives.
Quality Enhancement	Parking facilities	Urban environment, motor vehicles, and people	Enhancing the quality of the environment and serving people, and meeting parking needs	Guiding the development of urban parking, focusing on the environmental quality of parking facilities, and building a harmonious relationship between the environment, people, and vehicles.

**Table 2:** Parking Facility Quality Index Evaluation Index System.

	Evaluation Dimensions	Evaluation Content	Evaluation Indicators
Quality Index	Pedestrian Experience	Impact on pedestrian space	Number of entrances/exits
			Distance between entrances and exits
			Number of facilities
			Scale of demand
	Landscape environment	Impact on environmental space	Number of entrances/exits
			Distance between entrances and exits
			Number of facilities
			Facility type
	Functional requirements	Matching with parking demand	Number of supplies
			Number of demands

**Table 3:** Information on 9 typical parking facilities.

Type of Parking Facilities	Location	Parking Facility Environment	Entrance/Exit Location Information	Photo
building-assigned parking plenty	Jiefangbei–Chaotianmen Area	The parking lot is located northwest of the Jiefangbei business district and is surrounded by mainly commercial areas.	There is one exit and one entrance for the Kui Xing Lou parking lot, which is located on the first floor and connected to the road, about 30 m away from the Red Line on City Road.	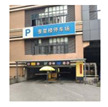
building-assigned parking plenty	Daping Commercial Area	The surrounding area of the car park is mainly commercial and bears the public parking needs of the Daping business district.	The complex’s parking garage, with an entrance and exit, is adjacent to the city’s commercial and residential land.	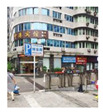
building-assigned parking plenty	Three Gorges Cultural Area	Access to the car park is via an approximately 50-m-long access road, which connects external and internal traffic.	Parking entrances and exits are established at the same location, via two-way roads.	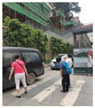
urban public parking plenty	Three Gorges Cultural Area	The parking lot is surrounded by People’s Square, the Three Gorges Museum, Tai gu Square, and additional leisure and entertainment areas.	The parking lot of Chongqing Municipal People’s Auditorium has surface and underground parking. Ground-level parking entrances line the sides of the plaza, and underground parking entrances and exits line the roads to and from the plaza.	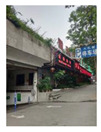
urban public parking plenty	Jiefangbei–Chaotianmen Area	The parking lot is located near the Hongyadong Scenic Area, which can solve the parking demand during the peak tourist season.	The parking lot is located in the heart of Old Town and has only one external entrance/exit, which will have an impact on the city’s dynamic traffic during peak hours.	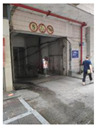
urban public parking plenty	Riverside Tourist Area	The parking lot is located in an open area in the Riverfront area, adjacent to City Road and surrounded by numerous businesses.	The parking lot is equipped with an entrance and exit, and vehicle access has an impact on the city’s dynamic traffic.	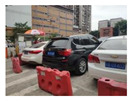
on-street parking plenty	Daping Commercial Area	Parking spaces are located along the street, in residential areas on the periphery of commercial areas.	Parking lots were set up along both sides of the road, along with managers. Vehicle parking has an impact on dynamic traffic and affects the walking line of sight for pedestrians.	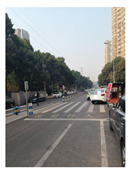
on-street parking plenty	Jiefangbei–Chaotianmen Area	The parking lot is mainly surrounded by People’s Park and the Jiefangbei shopping area.	Parking spaces were established along the parkway, with one side of the parkway ending next to Xinhua Road and the remaining side next to People’s Park.	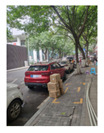
on-street parking plenty	Red Rock Spirit Cultural Area	The car park is located outside the residential area to address parking issues for some residents.	Parking lots were set up along both sides of the road, along with managers. Vehicle parking has an impact on dynamic traffic and affects the walking line of sight for pedestrians.	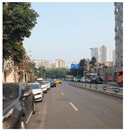

**Table 4:** Environmental impact assignment of parking facility types.

Type of Parking Facilities	Environmental Impact Assignments
Surface Parking	1–3
Underground Parking	3–8
Parking Building	5–9

Source: Redrawn from “Study on Urban Parking Facilities Planning Methodology”.

**Table 5:** Evaluation result score range of urban parking facilities.

Score Range	Description of Evaluation Results
0 to 2 points	Insufficient supply of parking spaces to meet parking demand
2 to 4 points	Parking needs are basically met, but parking is inconvenient in special cases
4 to 6 points	Basic functions to meet basic urban parking needs
6 to 8 points	Meet the basic function of parking needs, while the impact on pedestrian travel and urban landscape environment is greater
8 to 10 points	Urban parking demand is met with low impact on pedestrian travel and urban landscape environment

**Table 6:** (**a**): Basic information of interviewees (driver). (**b**): Basic information of interviewees (pedestrians).

Items	Contents	Number	Proportion (%)
**a**			
Gender	Male	77	71.3
	Female	31	28.7
Age	≤22	3	2.7%
	23~35	63	58.3%
	36~50	39	36%
	51~60 years old	2	2%
	≥60	1	1%
Career	Government Employee	8	7.4%
	Corporate or business unit personnel	70	64.8%
	Retirees	2	1.9%
	Students	19	17.6%
	Freelance	7	6.4%
	Others	2	1.9%
Fixed parking spaces (residence)	Yes	91	84.3%
	No	17	15.7%
Fixed parking space (unit)	Yes	75	69.4%
	No	33	30.6%
**b**			
Gender	Male	58	58%
	Female	42	42%
Age	≤22	10	10%
	23~35	48	48%
	36~50	25	25%
	51~60 years old	12	12%
	≥60	5	5%
Career	Government Employee	6	6%
	Corporate or business unit personnel	69	69%
	Retirees	2	2%
	Students	15	15%
	Freelance	8	8%
	Others	0	0%
Walking hours (per day)	<30 min	18	18%
	30 min~1 h	47	47%
	1 h~2 h	32	32%
	≥2 h	3	3%
Number of parking lots (pedestrians passing by)	0	3	3%
	1	26	26%
	2	45	45%
	≥3	26	26%
Number of parking entrances and exits (pedestrians passing by)	0	2	2%
	1	22	22%
	2	39	39%
	3	25	25%
	≥4	12	12%

**Table 7:** Results of an analysis of drivers’ factors in choosing parking facilities.

		Respondents Were Concerned about the Environmental Factors of Parking Facilities.	Respondents’ Rationale for Choosing Parking Facilities
Respondents’ top factors for choosing a parking facility	Correlation coefficient	−0.243 *	0.207 *
	*p*-value	0.010	0.029

* *p* < 0.05.

**Table 8:** Results of the analysis of factors affecting pedestrians by parking facilities.

		Availability of Parking Signs	The number of Parking Lot Entrances and Exits the Respondent Passed on His/Her Walk	The Number of Parking Lots the Respondent Passed on His/Her Walk
Whether parking facilities have an impact on walking	Correlation coefficient	−0.324 *	0.437 *	0.513 **
	*p*-value	0.021	0.028	0.008

* *p* < 0.05 ** *p* < 0.01.

## Data Availability

Not applicable.
